# Clozapine-related obsessive–compulsive symptoms and their impact on wellbeing: a naturalistic longitudinal study

**DOI:** 10.1017/S003329172100492X

**Published:** 2023-05

**Authors:** Katherine Parkin, Shanquan Chen, Marjan Biria, James Plaistow, Helen Beckwith, Isaac Jarratt-Barnham, Nuria Segarra, Yulia Worbe, Naomi A. Fineberg, Rudolf N. Cardinal, Trevor W. Robbins, Emilio Fernandez-Egea

**Affiliations:** 1 Cambridgeshire and Peterborough NHS Foundation Trust, Cambridge, UK; 2 Department of Public Health and Primary Care, University of Cambridge, Cambridge, UK; 3 Department of Psychiatry, University of Cambridge, Cambridge, UK; 4 Behavioural and Clinical Neuroscience Institute, University of Cambridge, Cambridge, UK; 5 Department of Psychology, University of Cambridge, Cambridge, UK; 6 Sorbonne Université, Paris 05, France; 7 Department of Neurophysiology, Saint-Antoine Hospital, Paris, France; 8 INSERM U 1127, CNRS UMR 7225, Institute du Cerveau et de la Moelle Epinière, Paris, France; 9 Hertfordshire Partnership University NHS Foundation Trust, Welwyn Garden City, UK; 10 School of Life and Medical Sciences, University of Hertfordshire, Hatfield, UK; 11 University of Cambridge School of Clinical Medicine, Addenbrooke's Hospital, Cambridge, UK

**Keywords:** Clozapine, obsessive–compulsive disorder (OCD), obsessive–compulsive symptoms (OCS), schizophrenia, wellbeing

## Abstract

**Background:**

Obsessive–compulsive symptoms (OCS) are commonly associated with clozapine treatment but are frequently overlooked by clinicians despite their potential impact on patients' quality of life. In this study, we explored whether OCS severity impacted subjective wellbeing and general functioning, independently of depressive and psychotic symptoms.

**Methods:**

We used anonymised electronic healthcare records from a large cohort of patients who were treated with clozapine and assessed annually for OCS, wellbeing, general functioning, and psychopathology using standardised scales as part of routine clinical practice. We used statistical mixed linear model techniques to evaluate the longitudinal influence of OCS severity on wellbeing and general functioning.

**Results:**

A total of 184 patients were included, with 527 face-to-face assessments and 64.7% evaluated three or more times. Different linear mixed models demonstrated that OCS in patients treated with clozapine were associated with significantly worse wellbeing scores, independently of depression and psychotic symptoms, but OCS did not impair general functioning. Obsessional thinking and hoarding behaviour, but not compulsions, were significantly associated with the impact on wellbeing, which may be attributable to the ego-syntonic nature of the compulsions.

**Conclusions:**

Given the frequent occurrence of OCS and their negative impact on wellbeing, we encourage clinicians to routinely assess and treat OCS in patients who are taking clozapine.

## Introduction

People with schizophrenia diagnoses experience lower subjective wellbeing compared to the general population (Maat, Fett, Derks, & Investigators, 2012; Ritsner et al., 2000). This difference is especially pronounced in those with ‘deficit subtypes’ of schizophrenia (Sum, Tay, Sengupta, & Sim, 2018). However, the determinants of subjective wellbeing in patients with schizophrenia are still unclear.

Depressive (van Rooijen et al., 2019) and psychotic symptoms (Brown, Mezquida, & Fernandez-Egea, 2016) are known to reduce wellbeing in this patient group. Recently, we found that medication-induced obsessive–compulsive symptoms (OCS) might also have a detrimental effect on wellbeing (Biria et al., 2019). OCS induced by antipsychotics are common and primarily associated with antipsychotics with strong antiserotonergic activity (Grillault Laroche & Gaillard, 2016; Swets et al., 2014), such as clozapine and olanzapine (Poyurovsky et al., 2001; Schirmbeck et al., 2011; Schirmbeck & Zink, 2012). Estimates of OCS prevalence for individuals using these medications are broad, ranging from 11% to 64% for symptoms only and from 0% to 37.5% for obsessive–compulsive disorder (OCD) (Swets et al., 2014), depending on the methodology used. Nevertheless, they are higher than in the general population, in which the lifetime prevalence of OCD is 1.6% (Kessler et al., 2005). In previous work (Fernandez-Egea, Worbe, Bernardo, & Robbins, 2018), we found that 47% of patients treated with clozapine (40% of whom were treated with clozapine for over 20 years) developed significant OCS, measured by the Obsessive–Compulsive Inventory – Revised (OCI-R; Foa et al., 2002). This proportion is three times higher than in patients diagnosed with schizophrenia who are not treated with clozapine (Swets et al., 2014). Moreover, the percentage of patients developing clinically significant compulsive behaviour was significantly correlated with the duration of clozapine treatment (Fernandez-Egea et al., 2018).

In a recent study, we found that in patients treated with clozapine, those with new-onset OCS experienced significantly lower subjective wellbeing compared to those without OCS (Biria et al., 2019). However, this study was cross-sectional, using a small sample size (*n* = 85; 56 with OCS and 29 without OCS) and did not account for other factors known to impact wellbeing, such as depression (van Rooijen et al., 2019) and psychotic symptoms (Brown et al., 2016). Gürcan, Şenol, Yağcıoğlu, and Ertuğrul (2021) recently explored the impact of clozapine-induced OCS in patients with schizophrenia, along with the clinical risk factors and phenomenology of OCS. They found that the severity of OCS positively correlated with the severity of depression, ‘positive’ symptoms (i.e. psychosis), and general psychopathology. Furthermore, the severity of OCS correlated with decreased functioning, as measured by the World Health Organization Disability Assessment Schedule II (WHO-DAS II). Their results suggest that both the severity of OCS and schizophrenia psychopathology decreased people's level of functioning. As noted by Gürcan et al. (2021), studies using a longitudinal design would be needed to explore this further.

In the present study, we aimed to explore whether OCS severity affects both subjective wellbeing and general functioning, independently of depression and psychosis. We used a naturalistic longitudinal design using electronic healthcare records from a large cohort of patients treated with clozapine who were assessed annually for OCS and wellbeing (both since 2016), and general functioning, depressive, and psychotic symptoms (all since 2012), as part of routine clinical practice. We used statistical mixed linear modelling techniques to evaluate the longitudinal influence of OCS severity on wellbeing and general functioning.

## Method

### Study design and participants

We conducted a retrospective analysis using the electronic records of Cambridgeshire and Peterborough NHS Foundation Trust (CPFT). CPFT is the primary public mental health care provider for a population of approximately 860 000 people in a mixed urban/rural area (including the cities and towns of Cambridge, Peterborough, Huntingdon, and Ely) in the East of England, UK. We used the Clinical and Research Database (CRD) for Persistent Schizophrenia, under NHS Research Ethics approvals (ref. 18/EE/0239; NHS Health Research Authority, 2021). This database contains anonymised routine clinical data from the CPFT Clozapine Clinic (see Fernandez-Egea et al., 2021). All clinical assessments in the CRD were performed by an experienced psychiatrist (EFE) or self-rated by the patient during routine clinical appointments. The data were collected between 24th August 2012 and 1st August 2020.

The CRD data included a total of 2560 assessments of 241 patients taking clozapine. As part of the care plan approach for everyone receiving care at the CPFT Clozapine Clinic, all individuals were asked to complete measures of psychotic and depressive symptoms, wellbeing, general functioning, and OCS on an annual basis. For this study, only those face-to-face assessments in which the OCI-R was completed were included. The final sample used data from 184 individuals (76% of the total sample); see Table 1 for details. Some completed these assessments on multiple occasions, producing a total of 527 data points. The date at which each patient completed the OCI-R for the first time constituted the baseline for that individual. The date at which an individual's last CRD data was recorded defined the follow-up duration.

**Table 1.** Sociodemographic and clinical description of the 184 patients included in the study

Variables	Number [%] or Mean (s.d.)
Individual level:	
Age (baseline)	45.9 (10.9)
Age at first episode psychosis	22.8 (7.4)
Gender (=male)	147 [79.9%]
Follow-up (months)	26.5 (14.6)
Number of face-to-face assessments during study period	
1	30 [16.3%]
2	35 [19%]
3	64 [34.8%]
4	40 [21.7%]
5	15 [8.2%]
Assessment level:	
Smoking (cigarettes/day)	5.86 (9.6)
Alcohol (units/week)	5.9 (14.5)
Duration of clozapine treatment (years)	16.31 (9.8)
Clozapine dose (in mg/day)	318.0 (141.9)
Wellbeing (SWEMWBS) score (corrected value)	22.07 (4.44)
Obsessive–compulsive symptoms (OCI-R) overall score	18.95 (13.4)
OCI-R Washing	1.72 (2.38)
OCI-R Obsessional thinking	4.35 (3.51)
OCI-R Hoarding	3.2 (2.85)
OCI-R Ordering	2.62 (2.83)
OCI-R Checking	4.72 (3.57)
OCI-R Mental neutralising	2.35 (2.9)
Severity of schizophrenia symptoms (CGI-SCH) overall score	3.16 (1.12)
CGI-SCH Depressive symptoms	1.57 (0.98)
CGI-SCH Positive/psychotic symptoms	2.46 (1.47)
CGI-SCH Negative symptoms	2.79 (1.28)
CGI-SCH Cognitive symptoms	2.48 (0.94)
General functioning (GAF) score	73.7 (13.4)

Data are presented as number [percentage] for categorical variables and mean (s.d.) for continuous variables. SWEMWBS: Short Warwick–Edinburgh Mental Wellbeing Scale. OCI-R: Obsessive–Compulsive Inventory – Revised. CGI-SCH: Clinical Global Impression – Schizophrenia. GAF: Global Assessment of Functioning Scale.

### Outcomes and measures

The first outcome was mental wellbeing, measured by the Short Warwick–Edinburgh Mental Wellbeing Scale (SWEMWBS; Stewart-Brown et al., 2009). This scale is valid and reliable for measuring mental wellbeing in diverse UK populations and projects (Stewart-Brown et al., 2011). The SWEMWBS is a self-report 7-item scale which asks about thoughts and feelings over the past 2 weeks, pertaining to items such as optimism, usefulness, thinking clearly, and closeness to others. Respondents are asked to rate how often they have experienced each statement over the last 2 weeks, on a 5-point scale ranging from ‘none of the time’ to ‘all of the time’. Responses are then summed, with higher scores indicating greater wellbeing. The corrected score was used for our analyses (Stewart-Brown et al., 2009).

The second outcome was general functioning, measured by the Global Assessment of Functioning Scale (GAF), a revision of the original Global Assessment Scale (GAS; Endicott, Spitzer, Fleiss, & Cohen, 1976). This scale is also used to rate how serious a mental illness may be (Schwartz, 2007) and has been recommended for routine clinical use (Salvi, Leese, & Slade, 2005). The GAF measures how much a person's symptoms affect their day-to-day life on a scale of 1–100, with higher scores indicating greater functioning. In this study, the rater was always the treating clinician. The rater is asked to consider psychological, social, and occupational functioning on a hypothetical continuum of mental health, over the past month, where the lowest level of functioning is chosen; the rater is asked to ignore impairment in functioning due to physical or environmental limitations. As such, the GAF covers a range from severe psychopathology to positive mental health, and gives an overall indication of how patients are doing (Aas, 2010).

The severity of OCS was measured by the Obsessive–Compulsive Inventory – Revised (OCI-R; Foa et al., 2002). The OCI-R is a self-report measure with 18 items comprising six subscales: obsessional thinking, washing, checking, ordering, hoarding behaviours, and mental neutralising. Respondents are asked to rate how much they have been distressed or bothered by various experiences over the last month, on a 5-point scale ranging from ‘not at all’ to ‘extremely’. The responses are then summed to give a total score in the range of 0–72, with higher scores indicating greater severity of OCS.

The overall severity of symptoms was measured by the Clinical Global Impression – Schizophrenia scale (CGI-SCH; Haro et al., 2003). The CGI-SCH scale, derived from the Clinical Global Impression (CGI) scale, is a clinician-rated measure which measures illness severity. The CGI-SCH has been used for efficacy and effectiveness studies in schizophrenia, and has been shown to be sensitive to change (Haro et al., 2003). It comprises four symptom domains (‘positive’/psychotic symptoms, ‘negative’ symptoms, depressive symptoms, and cognitive symptoms), and an overall severity domain. The rating is based on the last week and marked on a 7-point scale ranging from ‘0 = absent’ to ‘6 = extreme’, with higher scores indicating more severe symptoms.

We also collated other factors of interest for this study, including (1) sociodemographic information: age at baseline (years), gender (male *v.* female); (2) habits such as smoking (cigarettes per day) and alcohol use (units per week); and (3) key psychiatric information: age of the first episode of psychosis (FEP), duration of clozapine use, and clozapine dose.

### Statistical analysis

Linear mixed models, with intercept as a (per-subject) random variable, were used to assess the longitudinal effect of changes in OCI-R and CGI-SCH upon the outcomes of interest. Linear mixed modelling is a robust statistical technique for longitudinal analysis with repeated measures. To test associations of interest, we performed a hierarchical approach to fit the data. Taking the first dependent variable, mental wellbeing, as an example, we first fitted the data using the overall OCI-R score (or subscores) and overall CGI-SCH score (or subscores) as the independent variables (models 1, 2, 4, and 6 in Table 2). Next, we added interaction terms between the overall OCI-R score and CGI-SCH subscores (models 3, 5, and 7 in Table 2). Of note, we only added interaction terms for OCI-R subscores or CGI-SCH subscores when they were associated with statistically significant main effects in the first step. All models controlled for age at baseline, age at FEP, gender, smoking (cigarettes per day), alcohol use (units per week), length of clozapine treatment (in years), and clozapine dose (in mg/day). Confound variables to control for were chosen *a priori* based on our earlier research (Biria et al., 2019; Fernandez-Egea et al., 2018), which identified that clozapine dose and treatment duration were important for OCS, as were variables that influenced the individual's level of clozapine, such as smoking habits, alcohol use, and gender. Age at baseline and age at FEP were included as traditional demographic and psychiatric information as per convention in this field of research. The same approach as described above was used with the other dependent variable, general functioning.

**Table 2.** Hierarchical linear regression examining the effect of psychopathology on wellbeing (SWEMWBS) score

	Model 1		Model 2		Model 3		Model 4		Model 5		Model 6		Model 7	
	Coefficient (95% CI)	*p* value	Coefficient (95% CI)	*p* value	Coefficient (95% CI)	*p* value	Coefficient (95% CI)	*p* value	Coefficient (95% CI)	*p* value	Coefficient (95% CI)	*p* value	Coefficient (95% CI)	*p* value
Severity of obsessive–compulsive symptoms (OCI-R scores):														
Overall	**−0.09 (−0.12 to −0.06)**	**<0.001**	**−0.08 (−0.11 to −0.05)**	**<0.001**	**−0.11 (−0.18 to −0.05)**	**0.001**								
Subscores														
Washing							0.11 (−0.08 to 0.30)	0.242	0.11 (−0.08 to 0.30)	0.2414	0.08 (−0.11 to 0.26)	0.402	0.09 (−0.09 to 0.28)	0.326
Obsessional thinking							**−0.38 (−0.51 to −0.24)**	**<0.001**	−0.24 (−0.61 to 0.12)	0.185	**−0.29 (−0.43 to −0.15)**	**<0.001**	**−0.36 (−0.63 to −0.09)**	**0.009**
Hoarding							**−0.22 (−0.38 to −0.07)**	**0.005**	**−0.41 (−0.83 to 0.00)**	**0.0497**	**−0.19 (−0.34 to −0.04)**	**0.013**	**−0.37 (−0.67 to −0.06)**	**0.018**
Ordering							−0.11 (−0.27 to 0.05)	0.19	−0.11 (−0.27 to 0.05)	0.1959	−0.12 (−0.28 to 0.04)	0.129	−0.12 (−0.28 to 0.04)	0.129
Checking							−0.03 (−0.17 to 0.10)	0.616	−0.03 (−0.16 to 0.10)	0.6388	−0.05 (−0.18 to 0.08)	0.444	−0.05 (−0.18 to 0.08)	0.445
Mental neutralising							0.12 (−0.03 to 0.28)	0.121	0.12 (−0.04 to 0.27)	0.1428	0.12 (−0.03 to 0.28)	0.108	0.12 (−0.04 to 0.27)	0.141
Severity of schizophrenia symptoms (CGI-SCH scores):														
Overall	**−0.69 (−1.05 to −0.34)**	**<0.001**					**−0.62 (−0.96 to −0.27)**	**0.001**	**−0.64 (−1.19 to −0.09)**	**0.0219**				
Subscores														
Depressive symptoms			**−0.74 (−1.10 to −0.38)**	**<0.001**	**−1.19 (−1.89 to −0.48)**	**0.001**					**−0.61 (−0.97 to −0.25)**	**0.001**	**−1.23 (−1.96 to −0.50)**	**0.001**
Positive/psychotic symptoms			**−0.51 (−0.79 to −0.22)**	**0.001**	**−0.55 (−1.02 to −0.08)**	**0.022**					**−0.43 (−0.72 to −0.14)**	**0.004**	**−0.50 (−0.97 to −0.03)**	**0.039**
Negative symptoms			−0.25 (−0.60 to 0.10)	0.163	−0.26 (−0.61 to 0.09)	0.149					−0.28 (−0.62 to 0.07)	0.118	−0.29 (−0.64 to 0.06)	0.101
Cognitive symptoms			−0.23 (−0.66 to 0.20)	0.295	−0.21 (−0.65 to 0.22)	0.336					−0.23 (−0.66 to 0.20)	0.301	−0.21 (−0.64 to 0.22)	0.346
Interaction between OCS and CGI-SCH:														
OCI-R (overall): CGI-SCH (depressive symptoms)					0.02 (−0.01 to 0.05)	0.159								
OCI-R (overall): CGI-SCH (positive/psychotic symptoms)					0.00 (−0.02 to 0.02)	0.894								
OCI-R (obsessional thinking): CGI-SCH (overall)									−0.04 (−0.14 to 0.06)	0.4337				
OCI-R (hoarding): CGI-R (overall)									0.06 (−0.06 to 0.18)	0.3258				
OCI-R (obsessional thinking): CGI-SCH (depressive symptoms)													0.05 (−0.07 to 0.17)	0.399
OCI-R (obsessional thinking): CGI-SCH (positive/psychotic symptoms)													0.00 (−0.09 to 0.08)	0.968
OCI-R (hoarding): CGI-SCH (depressive symptoms)													0.07 (−0.04 to 0.18)	0.223
OCI-R (hoarding): CGI-SCH (positive/psychotic symptoms)													0.02 (−0.07 to 0.11)	0.645
Covariates:														
Age at baseline	**0.07 (0.01 to 0.12)**	**0.017**	**0.06 (0.00 to 0.11)**	**0.04**	**0.06 (0.00 to 0.11)**	**0.04**	**0.06 (0.01 to 0.12)**	**0.021**	**0.06 (0.01 to 0.12)**	**0.0196**	**0.06 (0.00 to 0.11)**	**0.034**	**0.06 (0.00 to 0.11)**	**0.036**
Age at FEP	**−0.09 (−0.16 to −0.01)**	**0.022**	−0.06 (−0.13 to 0.01)	0.11	−0.06 (−0.13 to 0.02)	0.127	−0.07 (−0.14 to 0.00)	0.067	−0.07 (−0.14 to 0.01)	0.073	−0.05 (−0.12 to 0.02)	0.165	−0.05 (−0.12 to 0.02)	0.196
Gender (=male)	0.46 (−0.84 to 1.77)	0.488	0.24 (−1.00 to 1.47)	0.708	0.24 (−0.99 to 1.47)	0.7	0.41 (−0.83 to 1.65)	0.514	0.40 (−0.84 to 1.64)	0.5272	0.27 (−0.92 to 1.47)	0.654	0.26 (−0.94 to 1.45)	0.675
Smoking (cigarettes/day)	−0.03 (−0.07 to 0.01)	0.197	−0.02 (−0.06 to 0.02)	0.323	−0.02 (−0.06 to 0.02)	0.354	−0.03 (−0.07 to 0.01)	0.134	−0.03 (−0.07 to 0.01)	0.1314	−0.02 (−0.06 to 0.02)	0.253	−0.02 (−0.06 to 0.02)	0.284
Alcohol (units/week)	0.00 (−0.02 to 0.03)	0.902	0.00 (−0.02 to 0.03)	0.899	0.00 (−0.02 to 0.02)	0.948	0.00 (−0.02 to 0.03)	0.743	0.01 (−0.02 to 0.03)	0.6876	0.00 (−0.02 to 0.03)	0.755	0.00 (−0.02 to 0.03)	0.747
Duration of clozapine use (years)	0.01 (−0.03 to 0.05)	0.595	0.02 (−0.02 to 0.06)	0.308	0.02 (−0.02 to 0.06)	0.322	0.01 (−0.03 to 0.04)	0.738	0.01 (−0.03 to 0.04)	0.7374	0.01 (−0.02 to 0.05)	0.458	0.02 (−0.02 to 0.05)	0.422
Clozapine dose (mg/day)	0.00 (0.00–0.00)	0.715	0.00 (0.00–0.00)	0.601	0.00 (0.00–0.00)	0.61	0.00 (0.00–0.00)	0.816	0.00 (0.00–0.00)	0.8108	0.00 (0.00–0.00)	0.7	0.00 (0.00–0.00)	0.721
Conditional *R*^2^:	0.634		0.639		0.648		0.627		0.627		0.638		0.645	

**Bold** indicates significant values, with associated p values in the column to the right-hand side. SWEMWBS: Short Warwick–Edinburgh Mental Wellbeing Scale; OCI-R: Obsessive–Compulsive Inventory – Revised; CGI-SCH: Clinical Global Impression – Schizophrenia.

Data was fitted by linear mixed models, with intercept as a (per-subject) random variable. A hierarchical approach was implemented by fitting the data using pairwise combination of overall OCI-R score (or subscores) and overall CGI-SCH score (or subscores) as the independent variables (models 1, 2, 4, and 6). Interaction terms were then added for OCI-R subscores or CGI-SCH subscores when they were associated with statistically significant main effects (models 3, 5, and 7).

Multicollinearity was tested using the variance inflation factor (VIF). VIF⩾10 indicates severe or serious multicollinearity (O'Brien, 2007). In this study, all models had a maximum VIF of 2.8, suggesting negligible multicollinearity. For each model, we also plotted a series of residual plots to visually check the other assumptions of linear models, including residuals *v.* fitted value to check the linear relationship assumptions, normal Q–Q to examine whether the residuals were normally distributed, and scale–location to check the homogeneity of variance of the residuals. The assumptions of linear models were not obviously violated. To measure the goodness-of-fit of the models, we report the conditional *R*^2^ in Tables 2 and 3.

**Table 3.** Hierarchical linear regression examining the effect of psychopathology on general functioning (GAF) score

	Model 1		Model 2		Model 3		Model 4		Model 5		Model 6	
	Coefficient (95%CI)	*p* value	Coefficient (95%CI)	*p* value	Coefficient (95%CI)	*p* value	Coefficient (95%CI)	*p* value	Coefficient (95%CI)	*p* value	Coefficient (95%CI)	*p* value
Severity of obsessive–compulsive symptoms (OCI-R scores):												
Overall	−0.05 (−0.13 to 0.02)	0.149	−0.04 (−0.11 to 0.04)	0.331	**−0.27 (−0.48 to −0.05)**	**0.01**						
Subscores												
Washing							−0.39 (−0.85 to 0.07)	0.094	**−0.48 (−0.92 to −0.04)**	**0.034**	−1.01 (−2.21 to 0.19)	0.1
Obsessional thinking							−0.28 (−0.61 to 0.05)	0.098	−0.02 (−0.36 to 0.31)	0.896	−0.03 (−0.37 to 0.30)	0.86
Hoarding							0.08 (−0.30 to 0.46)	0.687	0.30 (−0.06 to 0.67)	0.106	0.29 (−0.08 to 0.66)	0.12
Ordering							0.05 (−0.34 to 0.45)	0.789	−0.08 (−0.46 to 0.30)	0.679	−0.04 (−0.42 to 0.33)	0.82
Checking							0.17 (−0.15 to 0.49)	0.293	0.04 (−0.27 to 0.35)	0.796	0.04 (−0.27 to 0.35)	0.81
Mental neutralising							−0.05 (−0.43 to 0.34)	0.81	−0.09 (−0.46 to 0.27)	0.626	−0.05 (−0.42 to 0.32)	0.79
Severity of schizophrenia symptoms (CGI-SCH scores):	**−7.68 (−8.51 to −6.84)**	**<0.001**					**−7.59 (−8.43 to −6.74)**	**<0.001**				
Overall												
Subscores												
Depressive symptoms			**−2.13 (−2.98 to −1.28)**	**<0.001**	**−3.11 (−4.79 to −1.43)**	**<0.001**			**−2.27 (−3.13 to −1.40)**	**<0.001**	**−2.25 (−3.32 to −1.17)**	**<0.001**
Positive/psychotic symptoms			**−2.61 (−3.29 to −1.93)**	**<0.001**	**−2.68 (−3.80 to −1.56)**	**<0.001**			**−2.59 (−3.29 to −1.90)**	**<0.001**	**−2.30 (−3.12 to −1.48)**	**<0.001**
Negative symptoms			**−3.39 (−4.22 to −2.56)**	**<0.001**	**−4.16 (−5.43 to −2.89)**	**<0.001**			**−3.45 (−4.28 to −2.61)**	**<0.001**	**−3.89 (−4.88 to −2.91)**	**<0.001**
Cognitive symptoms			**−3.05 (−4.08 to −2.02)**	**<0.001**	**−3.29 (−5.00 to −1.59)**	**<0.001**			**−2.94 (−3.98 to −1.91)**	**<0.001**	**−3.04 (−4.29 to −1.79)**	**<0.001**
Interaction between OCS and CGI-SCH:												
OCI-R (overall): CGI-SCH (depressive symptoms)					0.04 (−0.03 to 0.10)	0.25						
OCI-R (overall): CGI-SCH (positive/psychotic symptoms)					0.00 (−0.04 to 0.05)	0.95						
OCI-R (overall): CGI-SCH (negative symptoms)					0.04 (−0.01 to 0.10)	0.11						
OCI-R (overall): CGI-SCH (cognitive symptoms)					0.02 (−0.05 to 0.09)	0.63						
OCI-R (washing): CGI-SCH (depressive symptoms)											−0.02 (−0.38 to 0.34)	0.9
OCI-R (washing): CGI-SCH (positive/psychotic symptoms)											−0.16 (−0.40 to 0.07)	0.18
OCI-R (washing): CGI-SCH (negative symptoms)											0.27 (−0.06 to 0.60)	0.11
OCI-R (washing): CGI-SCH (cognitive symptoms)											0.08 (−0.34 to 0.49)	0.72
Covariates:												
Age at baseline	−0.12 (−0.25 to 0.00)	0.053	**−0.15 (−0.27 to −0.03)**	**0.018**	**−0.15 (−0.27 to −0.03)**	**0.02**	**−0.15 (−0.28 to −0.02)**	**0.02**	**−0.17 (−0.30 to −0.05)**	**0.007**	**−0.17 (−0.30 to −0.05)**	**0.01**
Age at FEP	0.07 (−0.11 to 0.24)	0.45	0.13 (−0.04 to 0.30)	0.138	0.13 (−0.03 to 0.30)	0.12	0.09 (−0.08 to 0.26)	0.31	0.15 (−0.02 to 0.32)	0.094	0.15 (−0.01 to 0.32)	0.08
Gender ( = male)	−0.43 (−3.32 to 2.46)	0.771	−0.58 (−3.49 to 2.32)	0.694	−0.49 (−3.37 to 2.39)	0.74	−0.59 (−3.48 to 2.30)	0.689	−0.51 (−3.42 to 2.39)	0.729	−0.55 (−3.44 to 2.33)	0.71
Smoking (cigarettes/day)	**−0.15 (−0.25 to −0.05)**	**0.004**	**−0.10 (−0.20 to −0.01)**	**0.04**	**−0.10 (−0.20 to 0.00)**	**0.05**	**−0.15 (−0.25 to −0.05)**	**0.003**	**−0.10 (−0.20 to 0.00)**	**0.042**	**−0.11 (−0.21 to −0.01)**	**0.03**
Alcohol (units/week)	0.03 (−0.03 to 0.09)	0.291	0.03 (−0.03 to 0.09)	0.278	0.03 (−0.03 to 0.09)	0.28	0.03 (−0.03 to 0.09)	0.305	0.03 (−0.03 to 0.08)	0.375	0.03 (−0.03 to 0.09)	0.33
Duration of clozapine use (years)	0.01 (−0.08 to 0.10)	0.828	0.03 (−0.06 to 0.12)	0.489	0.03 (−0.06 to 0.11)	0.58	0.02 (−0.08 to 0.11)	0.725	0.04 (−0.05 to 0.13)	0.37	0.03 (−0.06 to 0.12)	0.5
Clozapine dose (mg/day)	0.00 (0.00–0.01)	0.29	0.01 (0.00–0.01)	0.08	0.01 (0.00–0.01)	0.08	0.00 (0.00–0.01)	0.222	0.01 (0.00–0.01)	0.054	0.01 (0.00–0.01)	0.07
Conditional R^2^:	0.730		0.774		0.774		0.728		0.774		0.773	

**Bold** indicates significant values, with associated p values in the column to the right-hand side. GAF: Global Assessment of Functioning Scale; OCI-R: Obsessive–Compulsive Inventory – Revised; CGI-SCH: Clinical Global Impression – Schizophrenia.

Data was fitted by linear mixed models, with intercept as a (per-subject) random variable. A hierarchical approach was implemented by fitting the data using pairwise combination of overall OCI-R score (or subscores) and overall CGI-SCH score (or subscores) as the independent variables (models 1, 2, 4, and 5). Interaction terms were then added for OCI-R subscores or CGI-SCH subscores when they were associated with statistically significant main effects (models 3 and 6).

All statistical analyses were performed using R (version 3.5.0; R Core Team, 2018), with the packages dplyr (1.0.2; Wickham, François, Henry, & Müller, 2020), lmerTest (3.1-2; Kuznetsova, Brockhoff, & Christensen, 2017), car (3.0-3; Fox & Weisberg, 2019), and MuMIn (1.43.17; Bartoń, 2020; adapted from Nakagawa, Johnson, & Schielzeth, 2017).

## Results

A total of 184 individuals were included in the study, with a total of 527 assessments in which OCI-R, SWEMWBS and GAF were completed on the same day. With regards to demographic information, 79.9% (*n* = 147) of the sample were male and the mean age of individuals was 45.9 years, with a standard deviation (s.d.) of 10.9 years. With regards to psychiatric history, the mean age of the first episode of psychosis was 22.8 years (s.d. = 7.4 years). With regards to current clinical status and clozapine treatment, the severity of schizophrenia symptoms overall score was 3.16 (s.d. = 1.12), the mean duration of clozapine treatment was 16.31 years (s.d. = 9.8 years) and the mean dose of clozapine was 318.0 mg/day (s.d. = 141.9 mg/day). With regards to outcome measures of interest, the mean OCI-R total score was 18.95 (s.d. = 13.4), the mean wellbeing corrected score was 22.07 (s.d. = 4.44) and the mean general functioning score was 73.7 (s.d. = 13.4). 41% of OCI-R scales scored 21 or more points, which is typically the threshold used to indicate OCD. For full details, see Table 1, which summarises the main sociodemographic information and clinical variables.

The influence of psychopathology, including OCS, on subjective wellbeing is shown in Table 2. Model 1 indicated that a 1 unit increase in OCS was associated with a 0.09 unit decrease in wellbeing. Model 2 and 6 indicated that the severity of depression and psychosis also influenced wellbeing. Model 4 and 6 indicated that obsessional thinking and hoarding behaviour domains were the determinant of effect on wellbeing. Model 3, 5, and 7 indicated that there were no interaction effects.

The influence of psychopathology, including OCS, on general functioning is shown in Table 3. Model 1 indicated that only general psychopathology, but not OCS, influenced general functioning. Subsequent models confirmed that all psychopathology domains measured with the CGI-SCH (psychotic, negative, depressive, and cognitive symptom severity) determined general functioning. No significant interaction effects were found.

## Discussion

We found that OCS experienced by patients treated with clozapine were significantly associated with worse wellbeing, but did not impair general functioning, as measured by self-report using the Short Warwick–Edinburgh Mental Wellbeing Scale and by clinician report using the Global Assessment of Functioning scale, respectively. The negative impact was detected even when controlling for depressive and psychotic symptoms. To the best of our knowledge, this study is the first to use longitudinal information to ascertain the impact of OCS on wellbeing and functioning.

The mean wellbeing score (22.07) in this study was similar to previous studies in this population (Brown et al., 2016) and lower than the mean for the general population (23.7 for men, 23.2 for women) (Ng Fat, Scholes, Boniface, Mindell, & Stewart-Brown, 2017). For each 1-point increase in the total OCI-R score, we found an associated 0.09-point decrease in the wellbeing score. In a previous study, we found that almost half of patients treated with clozapine scored 21 or more points on the OCI-R (Fernandez-Egea et al., 2018). This would represent a 1.89 decrease in wellbeing, which is in the range of clinically relevant levels of change (Warwick Medical School, 2020). Our longitudinal data replicates and expands on previous results, in which patients with higher OCI-R scores experienced lower wellbeing (Biria et al., 2019). We also replicated work by van Rooijen et al. (2019) and Brown et al. (2016), in which depressive and psychotic symptoms impact on the wellbeing of people with schizophrenia. However, we found that OCS exert an additional detrimental effect, even accounting for this.

When different OCS domains were evaluated, only obsessional thinking and hoarding behaviours, but not checking behaviours, had a deleterious impact on wellbeing. The lack of statistically significant impact of checking behaviours on wellbeing could potentially be attributed to their ego-syntonic nature, thus not causing distress to the patient. This might contribute to the under-recognition of clozapine-related OCS, as patients might not complain about excessive checking (Mukhopadhaya et al., 2009). This reinforces the need for clinicians to routinely ask about excessive checking. Interestingly, we also found that OCS did not impact clinician-reported general functioning, which was mostly associated with psychosis and other symptom domains measured by the CGI-SCH. Once again, the emerging picture is of somewhat ‘invisible’ OCS, in which patients might not find them distressing and/or clinicians do not notice the impact, despite the deleterious effect on wellbeing.

Previous work by our team explored any potential differences between those individuals who experienced OCS and those who did not whilst taking clozapine (Biria et al., 2019); though OCS are more usefully viewed on a continuum than in a binary manner, the main significant differences that emerged between groups were higher clozapine dose and younger paternal age at birth in those with OCS compared to those who did not have OCS whilst taking clozapine.

Clozapine remains a gold-standard treatment option for individuals diagnosed with schizophrenia who have not previously benefited from trying two or more other antipsychotic medications (Taylor, 2017) and is prescribed because it can reduce symptom severity and improve functioning. However, these benefits must be balanced with an awareness that clozapine-related OCS (either exacerbation of pre-existing OCS or development of new-onset OCS) can impair patients' wellbeing. Our results highlight the need to measure not just symptomatology but also patients' quality of life or subjective wellbeing (Felce & Perry, 1995), a frequently forgotten but important goal in schizophrenia care (Engel, 1977). These findings are especially significant considering that Mukhopadhaya et al. (2009) found ~50% of patients taking clozapine had never been asked about OCS, illustrating that this is an under-recognised problem in clinical settings. While people with schizophrenia diagnoses may report lower levels of happiness on average than healthy controls, there is considerable heterogeneity within this population (Palmer et al., 2016). Palmer et al. (2016) found that levels of happiness in people with schizophrenia were significantly related to various positive psychosocial factors, such as lower perceived stress and higher levels of resilience, optimism, and personal mastery. As such, increasing wellbeing and happiness remains a valid and important treatment goal for this population and assessing and treating clozapine-related OCS should be a clinical goal.

This study has various strengths, as well as limitations. A particular strength is the scope and comprehensiveness of data collection in a real-world clinical setting. All measures were administered systematically to everyone receiving care from the CPFT Clozapine Clinic, not just those where OCS, wellbeing or general functioning had been identified as an issue. This allowed us, uniquely, to explore the relationship between OCS, wellbeing and general functioning in patients treated with clozapine. Furthermore, the study has the largest sample size and longest longitudinal follow-up to date for evaluating OCS in this patient group. This was a naturalistic study with the research being embedded in routine clinical practice; as such, if individuals were identified as having clinically meaningful symptoms, they were offered treatment for this, consisting of pharmacological and non-pharmacological options. Naturalistic studies offer the benefit of being ecologically valid and applicable in real-world settings but tend to lack the consistency of ‘pure’ research where variables can be more stringently controlled. Furthermore, although there was no control group in this study, patients had multiple assessments so acted as their own comparator, demonstrating the impact of OCS over time. We used linear mixed models, considered to be a robust statistical method for real-world longitudinal data (Garcia & Marder, 2017), in which missing data or inconsistent intervals of assessment are common. Another possible limitation relates to the outcome measures used in the study. Firstly, we cannot entirely rule out the possibility of unblinded bias in the clinician-rated GAF scale. In addition, we used the OCI-R, which is a self-rated symptom scale for OCD, and less informative than other longer or more detailed scales such as the Yale–Brown Obsessive Compulsive Scale (Y-BOCS; Goodman et al., 1989) or full Obsessive–Compulsive Inventory Scale (OCI; Foa, Kozak, Salkovskis, Coles, & Amir, 1998). These longer scales are used in research settings and it is debatable whether they could be embedded into routine clinical practice, from which our sample originates. Similarly, wellbeing can be measured using different scales. The short version of the WEMWBS, the SWEMWBS, has shown its validity when compared against the longer version (Stewart-Brown et al., 2009) and has been validated in people with schizophrenia (Vaingankar et al., 2017). It measures only aspects related to happiness (Brown et al., 2016) but has been recommended as a scale for routine clinical assessment in the UK (UK Department of Health, 2011). In any case, both OCI-R and SWEMWBS offer a reasonable balance of validity and time spent in clinical practice to screen and assess OCS and wellbeing. Finally, there are inherent limitations in the use of self-report measures, such as the potential for social desirability bias or responses being influenced by the clinician–patient relationship. However, measures were intentionally administered on an annual basis to minimise the possibility of learning effects or recall of previous responses influencing subsequent responses.

Future research could address a number of areas. In particular, perhaps because of the under-recognition of this issue, there is no agreement on how to treat clozapine-related OCS and improve wellbeing in this group of patients. Future research should evaluate which interventions are most effective for clozapine-related OCS, which might include pharmacological optimisation, medication combinations, and/or psychological therapies known to be effective for OCD, such as cognitive–behavioural therapy (CBT). Another valuable addition to the evidence base would be qualitative accounts of people's experiences of clozapine treatment, OCS, and the impact on wellbeing, to complement quantitative data. Moreover, future studies could seek to understand the impact of different reporting methods and the possible impact of the clinician–patient relationship when completing measures, such as the possibility of social desirability bias when patients complete self-report measures. Research may be enhanced by triangulating self-reported measures, clinician-reported measures, and reports from significant others.

In conclusion, we found that: (1) a substantial subset of this patient group present with OCS, which have previously been shown to be associated with clozapine treatment; and (2) clozapine-related OCS have a negative impact on patients' subjective wellbeing, independently of psychosis and depression severity. Considering the high incidence of OCS found, the fact that these symptoms are often overlooked by researchers and clinicians, and that patients might not spontaneously raise this as a concern, we hope this paper will encourage clinicians to assess clozapine-treated patients for OCS routinely and actively.

## References

[ref1] Aas, I. H. M. (2010). Global assessment of functioning (GAF): Properties and frontier of current knowledge. Annals of General Psychiatry, 9(1), 1–11. 10.1186/1744-859X-9-20. PMC2880316 20459646

[ref2] Bartoń, K. (2020). MuMIn: Multi-Model Inference. Retrieved from https://cran.r-project.org/package=MuMIn.

[ref3] Biria, M., Huang, F.-X., Worbe, Y., Fineberg, N. A., Robbins, T. W., & Fernandez-Egea, E. (2019). A cross-sectional study of impact and clinical risk factors of antipsychotic-induced OCD. European Neuropsychopharmacology: The Journal of the European College of Neuropsychopharmacology, 29(8), 905–913. 10.1016/j.euroneuro.2019.06.006. 31303266 PMC6689324

[ref4] Brown, J. E. H., Mezquida, G., & Fernandez-Egea, E. (2016). Well-being in clozapine-treated schizophrenia patients: The significance of positive symptoms. Comprehensive Psychiatry, 68, 140–146. 10.1016/j.comppsych.2016.04.009. 27234195

[ref5] Endicott, J., Spitzer, R. L., Fleiss, J. L., & Cohen, J. (1976). The global assessment scale: A procedure for measuring overall severity of psychiatric disturbance. Archives of General Psychiatry, 33(6), 766–771. 10.1001/archpsyc.1976.01770060086012. 938196

[ref6] Engel, G. L. (1977). The need for a new medical model: A challenge for biomedicine. Science (New York, N.Y.), 196(4286), 129–136. 10.1126/science.847460. 847460

[ref7] Felce, D., & Perry, J. (1995). Quality of life: Its definition and measurement. Research in Developmental Disabilities, 16(1), 51–74. 10.1016/0891-4222(94)00028-8. 7701092

[ref8] Fernandez-Egea, E., Chen, S., Jenkins, C., Turrion, C., Mitchell, S. P., Dodwell, D. J. F., … Cardinal, R. N. (2021). The effect of clozapine on self-reported duration of sleep and its interaction with 23 other medications: A 5-year naturalistic study. Journal of Clinical Psychopharmacology, 41(5), 534–539. 10.1097/jcp.0000000000001432. 34519455

[ref9] Fernandez-Egea, E., Worbe, Y., Bernardo, M., & Robbins, T. W. (2018). Distinct risk factors for obsessive and compulsive symptoms in chronic schizophrenia. Psychological Medicine, 48(16), 2668–2675. 10.1017/S003329171800017X. 29455687 PMC6236440

[ref10] Foa, E. B., Huppert, J. D., Leiberg, S., Langner, R., Kichic, R., Hajcak, G., & Salkovskis, P. M. (2002). The obsessive-compulsive inventory: Development and validation of a short version. Psychological Assessment, 14(4), 485–496. 12501574

[ref11] Foa, E. B., Kozak, M. J., Salkovskis, P. M., Coles, M. E., & Amir, N. (1998). The validation of a new obsessive–compulsive disorder scale: The obsessive–compulsive inventory. Psychological Assessment, 10(3), 206–214.

[ref12] Fox, J., & Weisberg, S. (2019). An R companion to applied regression (3rd ed.). Los Angeles, CA: Sage Publications Sage CA.

[ref13] Garcia, T. P., & Marder, K. (2017). Statistical approaches to longitudinal data analysis in neurodegenerative diseases: Huntington's disease as a model. Current Neurology and Neuroscience Reports, 17(2), 1–9. 10.1007/s11910-017-0723-4. 28229396 PMC5633048

[ref14] Goodman, W. K., Price, L. H., Rasmussen, S. A., Mazure, C., Fleischmann, R. L., Hill, C. L., … Charney, D. S. (1989). The Yale–Brown obsessive compulsive scale: I. Development, use, and reliability. Archives of General Psychiatry, 46(11), 1006–1011. 2684084 10.1001/archpsyc.1989.01810110048007

[ref15] Grillault Laroche, D., & Gaillard, A. (2016). Induced obsessive–compulsive symptoms (OCS) in schizophrenia patients under atypical 2 antipsychotics (AAPs): Review and hypotheses. Psychiatry Research, 246, 119–128. 27690134 10.1016/j.psychres.2016.09.031

[ref16] Gürcan, G., Şenol, Ş. H., Yağcıoğlu, A. E. A., & Ertuğrul, A. (2021). Clinical risk factors, phenomenology and the impact of clozapine-induced obsessive–compulsive symptoms. Psychiatry Research, 296, 113665. 10.1016/j.psychres.2020.113665. 33465593

[ref17] Haro, J. M., Kamath, S. A., Ochoa, S. O., Novick, D., Rele, K., Fargas, A., … Kharbeng, A. (2003). The clinical global impression–schizophrenia scale: A simple instrument to measure the diversity of symptoms present in schizophrenia. Acta Psychiatrica Scandinavica, 107(Suppl. 416), 16–23. 10.1034/j.1600-0447.107.s416.5.x 12755850

[ref18] Kessler, R. C., Berglund, P., Demler, O., Jin, R., Merikangas, K. R., & Walters, E. E. (2005). Lifetime prevalence and age-of-onset distributions of DSM-IV disorders in the national comorbidity survey replication. Archives of General Psychiatry, 62(6), 593–602. 10.1001/archpsyc.62.6.593. 15939837

[ref19] Kuznetsova, A., Brockhoff, P. B., & Christensen, R. H. B. (2017). LmerTest package: Tests in linear mixed-effects models. Journal of Statistical Software, 82(13), 1–26. 10.18637/jss.v082.i13.

[ref20] Maat, A., Fett, A.-K., Derks, E., & Investigators, G. (2012). Social cognition and quality of life in schizophrenia. Schizophrenia Research, 137(1–3), 212–218. 22406280 10.1016/j.schres.2012.02.017

[ref21] Mukhopadhaya, K., Krishnaiah, R., Taye, T., Nigam, A., Bailey, A. J., Sivakumaran, T., & Fineberg, N. A. (2009). Obsessive–compulsive disorder in UK clozapine-treated schizophrenia and schizoaffective disorder: A cause for clinical concern. Journal of Psychopharmacology, 23(1), 6–13. 10.1177/0269881108089582. 18515449

[ref22] Nakagawa, S., Johnson, P. C. D., & Schielzeth, H. (2017). The coefficient of determination R^2^ and intra-class correlation coefficient from generalized linear mixed-effects models revisited and expanded. Journal of the Royal Society Interface, 14(134), 20170213. 28904005 10.1098/rsif.2017.0213 PMC5636267

[ref23] Ng Fat, L., Scholes, S., Boniface, S., Mindell, J., & Stewart-Brown, S. (2017). Evaluating and establishing national norms for mental wellbeing using the short Warwick-Edinburgh Mental Well-being Scale (SWEMWBS): Findings from the Health Survey for England. Quality of Life Research: An International Journal of Quality of Life Aspects of Treatment, Care and Rehabilitation, 26(5), 1129–1144. 10.1007/s11136-016-1454-8. 27853963 PMC5376387

[ref24] NHS Health Research Authority. (2021). The Clinical and Research Database for Persistent Schizophrenia V1. Retrieved from https://www.hra.nhs.uk/planning-and-improving-research/application-summaries/research-summaries/the-clinical-and-research-database-for-persistent-schizophrenia-v1/.

[ref25] O'Brien, R. M. (2007). A caution regarding rules of thumb for variance inflation factors. Quality & Quantity, 41(5), 673–690.

[ref26] Palmer, B. W., Martin, A. S., Depp, C. A., Glorioso, D. K., Jeste, D. V, Diego, S., … Jolla, L. (2016). Wellness within illness: Happiness in schizophrenia. Schizophrenia Research, 159(1), 151–156. 10.1016/j.schres.2014.07.027.Wellness. PMC4928639 25153363

[ref27] Poyurovsky, M., Hramenkov, S., Isakov, V., Rauchverger, B., Modai, I., Schneidman, M., … Weizman, A. (2001). Obsessive–compulsive disorder in hospitalized patients with chronic schizophrenia. Psychiatry Research, 102(1), 49–57. 10.1016/s0165-1781(01)00238-4. 11368839

[ref28] R Core Team. (2018). R: A Language and Environment for Statistical Computing. Vienna, Austria: R Foundation for Statistical Computing. Retrieved from https://www.r-project.org.

[ref29] Ritsner, M., Modai, I., Endicott, J., Rivkin, O., Nechamkin, Y., Barak, P., … Ponizovsky, A. (2000). Differences in quality of life domains and psychopathologic and psychosocial factors in psychiatric patients. The Journal of Clinical Psychiatry, *61*(11), 880–889. 10.4088/JCP.v61n1113. 11105747

[ref30] Salvi, G., Leese, M., & Slade, M. (2005). Routine use of mental health outcome assessments: Choosing the measure. The British Journal of Psychiatry, 186(2), 146–152. 10.1192/bjp.186.2.146. 15684239

[ref31] Schirmbeck, F., Esslinger, C., Rausch, F., Englisch, S., Meyer-Lindenberg, A., & Zink, M. (2011). Antiserotonergic antipsychotics are associated with obsessive–compulsive symptoms in schizophrenia. Psychological Medicine, 41(11), 2361–2373. 10.1017/S0033291711000419. 21466748

[ref32] Schirmbeck, F., & Zink, M. (2012). Clozapine-induced obsessive-compulsive symptoms in schizophrenia: A critical review. Current Neuropharmacology, 10(1), 88–95. 22942882 10.2174/157015912799362724 PMC3286851

[ref33] Schwartz, R. C. (2007). Concurrent validity of the global assessment of functioning scale for clients with schizophrenia. Psychological Reports, 100(2), 571–574. 17564234 10.2466/pr0.100.2.571-574

[ref34] Stewart-Brown, S., Platt, S., Tennant, A., Maheswaran, H., Parkinson, J., Weich, S., … Clarke, A. (2011). The warwick-Edinburgh mental well-being scale (WEMWBS): A valid and reliable tool for measuring mental well-being in diverse populations and projects. Journal of Epidemiology & Community Health, 65(Suppl. 2), A38–A39.

[ref35] Stewart-Brown, S., Tennant, A., Tennant, R., Platt, S., Parkinson, J., & Weich, S. (2009). Internal construct validity of the Warwick-Edinburgh Mental Well-being Scale (WEMWBS): A Rasch analysis using data from the Scottish Health Education Population Survey. Health and Quality of Life Outcomes, 7(1), 1–8. 10.1186/1477-7525-7-15. 19228398 PMC2669062

[ref36] Sum, M. Y., Tay, K. H., Sengupta, S., & Sim, K. (2018). Neurocognitive functioning and quality of life in patients with and without deficit syndrome of schizophrenia. Psychiatry Research, 263, 54–60. 10.1016/j.psychres.2018.02.025. 29499447

[ref37] Swets, M., Dekker, J., van Emmerik-van Oortmerssen, K., Smid, G. E., Smit, F., de Haan, L., & Schoevers, R. A. (2014). The obsessive–compulsive spectrum in schizophrenia, a meta-analysis and meta-regression exploring prevalence rates. Schizophrenia Research, 152(2–3), 458–468. 10.1016/j.schres.2013.10.033. 24361303

[ref38] Taylor, D. M. (2017). Clozapine for treatment-resistant schizophrenia: Still the gold standard? CNS Drugs, 31(3), 177–180. 10.1007/s40263-017-0411-6. 28258419

[ref39] UK Department of Health. (2011). *No health without mental health: Delivering better mental health outcomes for people of all ages*. Retrieved from https://assets.publishing.service.gov.uk/government/uploads/system/uploads/attachment_data/file/215811/dh_124057.pdf.

[ref40] Vaingankar, J. A., Abdin, E., Chong, S. A., Sambasivam, R., Seow, E., Jeyagurunathan, A., … Subramaniam, M. (2017). Psychometric properties of the short Warwick Edinburgh mental well-being scale (SWEMWBS) in service users with schizophrenia, depression and anxiety spectrum disorders. Health and Quality of Life Outcomes, 15(1), 1–11. 10.1186/s12955-017-0728-3. 28764770 PMC5539899

[ref41] van Rooijen, G., van Rooijen, M., Maat, A., Vermeulen, J. M., Meijer, C. J., Ruhé, H. G., … van Winkel, R. (2019). Longitudinal evidence for a relation between depressive symptoms and quality of life in schizophrenia using structural equation modeling. Schizophrenia Research, 208, 82–89. 10.1016/j.schres.2019.04.011. 31047723

[ref42] Warwick Medical School. (2020). Collect, score, analyse and interpret WEMWBS. Retrieved from https://warwick.ac.uk/fac/sci/med/research/platform/wemwbs/using/howto/.

[ref43] Wickham, H., François, R., Henry, L., & Müller, K. (2020). dplyr: A Grammar of Data Manipulation. Retrieved from https://dplyr.tidyverse.org/.

